# Dimethyl 2-nitro­biphenyl-4,4′-di­carboxyl­ate

**DOI:** 10.1107/S1600536814004218

**Published:** 2014-02-28

**Authors:** Vanessa C. M. Vieira, James A. Golen, Arnold L. Rheingold, David R. Manke

**Affiliations:** a Department of Chemistry and Biochemistry, University of Massachusetts Dartmouth, 285 Old Westport Road, North Dartmouth, MA 02747, USA; b Department of Chemistry, University of California, San Diego, 9500 Gilman Drive, La Jolla, CA 92093, USA

## Abstract

The title compound, C_16_H_13_NO_6_, exhibits a biphenyl unit with a dihedral angle between the two aryl rings of 56.01 (5)°. The two ester groups vary slightly from planarity, with ar­yl–ester dihedral angles of 4.57 (5) and 16.73 (5)°. The nitro group is turned from the aromatic unit with an ar­yl–nitro dihedral angle of 48.66 (4)°. In the crystal, mol­ecules are connected by weak C—H⋯O inter­actions, forming a three-dimensional network.

## Related literature   

For the synthesis of the title compound, see: Olkhovik *et al.* (2008 ▶). For coordination polymers featuring the 2-nitro­biphenyl-4,4′-di­carboxyl­ate linker, see: Jing *et al.* (2012 ▶).
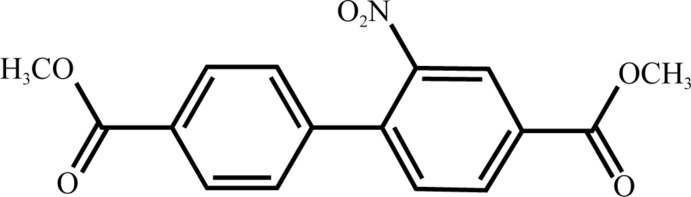


## Experimental   

### 

#### Crystal data  

C_16_H_13_NO_6_
*M* *_r_* = 315.27
Monoclinic, 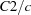

*a* = 20.3958 (17) Å
*b* = 8.3334 (6) Å
*c* = 18.9386 (14) Å
β = 118.342 (7)°
*V* = 2833.1 (4) Å^3^
*Z* = 8
Mo *K*α radiation
μ = 0.12 mm^−1^
*T* = 90 K
0.25 × 0.20 × 0.15 mm

#### Data collection  

Bruker APEXII CCD diffractometer
Absorption correction: multi-scan (*SADABS*; Bruker, 2005 ▶) *T*_min_ = 0.972, *T*_max_ = 0.983
19059 measured reflections
2918 independent reflections
2360 reflections with *I* > 2σ(*I*)
*R*_int_ = 0.029

#### Refinement  

*R*[*F* ^2^ > 2σ(*F* ^2^)] = 0.034
*wR*(*F* ^2^) = 0.097
*S* = 1.04
2918 reflections
210 parameters
H-atom parameters constrained
Δρ_max_ = 0.29 e Å^−3^
Δρ_min_ = −0.22 e Å^−3^

### 

Data collection: *APEX2* (Bruker, 2005 ▶); cell refinement: *SAINT* (Bruker, 2005 ▶); data reduction: *SAINT*; program(s) used to solve structure: *SHELXS97* (Sheldrick, 2008 ▶); program(s) used to refine structure: *SHELXL97* (Sheldrick, 2008 ▶); molecular graphics: *SHELXTL* (Sheldrick, 2008 ▶); software used to prepare material for publication: *SHELXTL*.

## Supplementary Material

Crystal structure: contains datablock(s) I, New_Global_Publ_Block. DOI: 10.1107/S1600536814004218/ff2127sup1.cif

Structure factors: contains datablock(s) I. DOI: 10.1107/S1600536814004218/ff2127Isup2.hkl

Supporting information file. DOI: 10.1107/S1600536814004218/ff2127Isup3.cml

CCDC reference:

Additional supporting information:  crystallographic information; 3D view; checkCIF report

## Figures and Tables

**Table 1:** Hydrogen-bond geometry (Å, °)

*D*—H⋯*A*	*D*—H	H⋯*A*	*D*⋯*A*	*D*—H⋯*A*
C4—H4*A*⋯O6^i^	0.95	2.50	3.3405 (16)	148
C13—H13*A*⋯O2^ii^	0.95	2.39	3.2435 (16)	150
C14—H14*A*⋯O4^iii^	0.95	2.59	3.3954 (16)	143

Symmetry codes: (i) 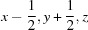; (ii) 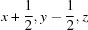; (iii) 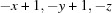.

**Fig. 1.**
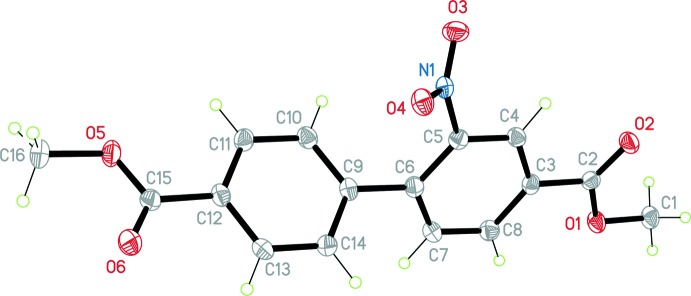
Molecular structure of the title compound, showing the atom labelling scheme. Displacement ellipsoids are drawn at the 50% probability level. H atoms are presented as spheres of arbitrary radius.

**Fig. 2.**
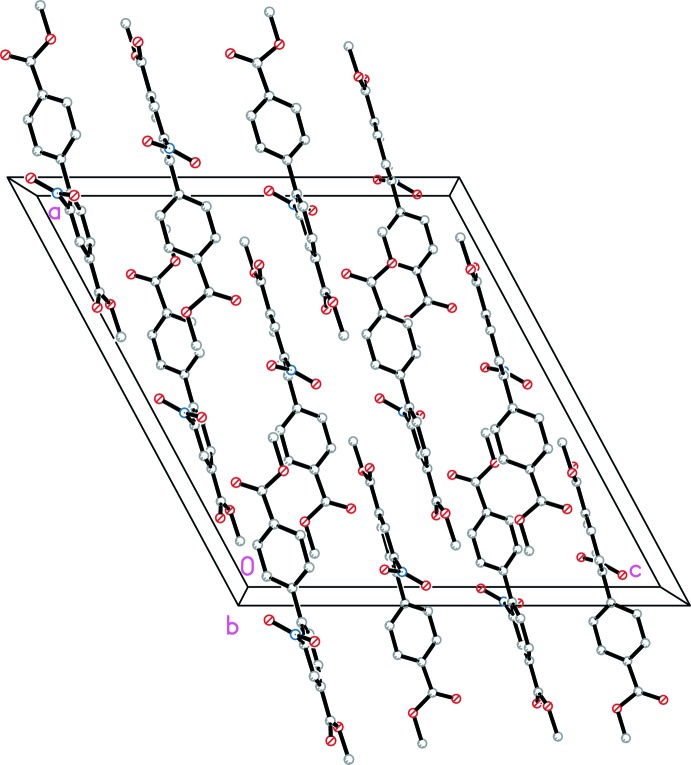
Molecular packing of the title compound.

## supplementary crystallographic information

### 1. Comment

Biphenyl-4,4'-dicarboxylate and its derivatives have become prevalent linkers in the preparation of metal-organic frameworks (MOFs). The ability to incorporate different functional groups into the pores of MOFs is one advantage of this class of materials. As a part of our efforts in this arena, we prepared the previously reported dimethyl 2-nitrobiphenyl-4,4'-dicarboxylate (Olkhovik *et al.* 2008) and report its structure herein.

The structure of the title compound is shown in Figure 1. The structure has a torsion angle of 56.01 (5)° between the two aryl rings. The ester groups vary slightly from the planes of the aromatic rings, with aryl-ester dihedral angles of 4.57 (5)° and 16.73 (5)°. The nitro group shows an aryl-nitro torsion angle of 48.66 (4)°. No π-π interactions were noted between the aromatic rings. The packing for the title compound is shown in Figure 2.

### 2. Experimental

The compound was prepared by literature procedure (Olkhovik *et al.* 2008). Crystals suitable for single-crystal X-ray analysis were grown by slow evaporation of an ethanol solution.

### 3. Refinement

Data corrected for absorption with *SADABS* (Bruker, 2005) and structure solved by direct methods (*SHELXS*) and all non-hydrogen atoms refined anisotropically by full matrix least squares on F^2^ (*SHELXL* (Sheldrick, 2008)). All hydrogen atoms were placed in calculated positions and then refined with riding model with C—H lengths of 0.95 Å for (CH) and 0.98 Å for (CH_3_) and with isotropic displacement parameters set to 1.20 and 1.50 times *U*_eq_ of the parent C atom.

### Crystal data

**Table:**

C_16_H_13_NO_6_	*F*(000) = 1312
*M**_r_* = 315.27	*D*_x_ = 1.478 Mg m^−^^3^
Monoclinic, *C*2/*c*	Mo *K*α radiation, λ = 0.71073 Å
Hall symbol: -C 2yc	Cell parameters from 7800 reflections
*a* = 20.3958 (17) Å	θ = 2.4–26.4°
*b* = 8.3334 (6) Å	µ = 0.12 mm^−^^1^
*c* = 18.9386 (14) Å	*T* = 90 K
β = 118.342 (7)°	Block, colorless
*V* = 2833.1 (4) Å^3^	0.25 × 0.20 × 0.15 mm
*Z* = 8	

### Data collection

**Table:**

Bruker APEXII CCD diffractometer	2918 independent reflections
Radiation source: fine-focus sealed tube	2360 reflections with *I* > 2σ(*I*)
Graphite monochromator	*R*_int_ = 0.029
φ and ω scans	θ_max_ = 26.5°, θ_min_ = 2.7°
Absorption correction: multi-scan (*SADABS*; Bruker, 2005)	*h* = −25→25
*T*_min_ = 0.972, *T*_max_ = 0.983	*k* = −10→10
19059 measured reflections	*l* = −23→23

### Refinement

**Table:**

Refinement on *F*^2^	Primary atom site location: structure-invariant direct methods
Least-squares matrix: full	Secondary atom site location: difference Fourier map
*R*[*F*^2^ > 2σ(*F*^2^)] = 0.034	Hydrogen site location: inferred from neighbouring sites
*wR*(*F*^2^) = 0.097	H-atom parameters constrained
*S* = 1.04	*w* = 1/[σ^2^(*F*_o_^2^) + (0.0512*P*)^2^ + 1.4779*P*] where *P* = (*F*_o_^2^ + 2*F*_c_^2^)/3
2918 reflections	(Δ/σ)_max_ < 0.001
210 parameters	Δρ_max_ = 0.29 e Å^−^^3^
0 restraints	Δρ_min_ = −0.22 e Å^−^^3^

### Special details

**Table:**

Geometry. All e.s.d.'s (except the e.s.d. in the dihedral angle between two l.s. planes) are estimated using the full covariance matrix. The cell e.s.d.'s are taken into account individually in the estimation of e.s.d.'s in distances, angles and torsion angles; correlations between e.s.d.'s in cell parameters are only used when they are defined by crystal symmetry. An approximate (isotropic) treatment of cell e.s.d.'s is used for estimating e.s.d.'s involving l.s. planes.
Refinement. Refinement of *F*^2^ against ALL reflections. The weighted *R*-factor *wR* and goodness of fit *S* are based on *F*^2^, conventional *R*-factors *R* are based on *F*, with *F* set to zero for negative *F*^2^. The threshold expression of *F*^2^ > σ(*F*^2^) is used only for calculating *R*-factors(gt) *etc*. and is not relevant to the choice of reflections for refinement. *R*-factors based on *F*^2^ are statistically about twice as large as those based on *F*, and *R*- factors based on ALL data will be even larger.

### Fractional atomic coordinates and isotropic or equivalent isotropic displacement parameters (Å^2^)

**Table:**

	*x*	*y*	*z*	*U*_iso_*/*U*_eq_	
O1	0.20578 (5)	0.35494 (11)	0.07049 (6)	0.0225 (2)	
O2	0.19389 (5)	0.61983 (11)	0.04893 (6)	0.0241 (2)	
O3	0.43918 (5)	0.91646 (12)	0.14067 (6)	0.0294 (3)	
O4	0.48248 (5)	0.82157 (11)	0.06479 (5)	0.0231 (2)	
O5	0.81617 (5)	0.57857 (12)	0.26263 (6)	0.0250 (2)	
O6	0.78697 (5)	0.47958 (13)	0.14129 (6)	0.0304 (3)	
N1	0.44911 (6)	0.80702 (13)	0.10372 (6)	0.0197 (3)	
C1	0.12899 (7)	0.35323 (17)	0.05505 (9)	0.0239 (3)	
H1A	0.1125	0.2420	0.0527	0.036*	
H1B	0.0974	0.4064	0.0038	0.036*	
H1C	0.1251	0.4101	0.0982	0.036*	
C2	0.23120 (7)	0.49999 (15)	0.06615 (8)	0.0186 (3)	
C3	0.31111 (7)	0.49867 (15)	0.08548 (7)	0.0182 (3)	
C4	0.34421 (7)	0.64598 (16)	0.08921 (7)	0.0179 (3)	
H4A	0.3169	0.7428	0.0807	0.022*	
C5	0.41786 (7)	0.64881 (16)	0.10550 (7)	0.0178 (3)	
C6	0.46139 (7)	0.51142 (16)	0.12059 (8)	0.0191 (3)	
C7	0.42667 (7)	0.36580 (16)	0.11802 (8)	0.0210 (3)	
H7A	0.4546	0.2693	0.1289	0.025*	
C8	0.35235 (7)	0.35825 (16)	0.10001 (8)	0.0206 (3)	
H8A	0.3297	0.2573	0.0976	0.025*	
C9	0.54141 (7)	0.51468 (16)	0.14053 (8)	0.0188 (3)	
C10	0.59328 (7)	0.60408 (16)	0.20508 (8)	0.0206 (3)	
H10A	0.5775	0.6654	0.2366	0.025*	
C11	0.66769 (7)	0.60418 (16)	0.22374 (8)	0.0201 (3)	
H11A	0.7030	0.6635	0.2685	0.024*	
C12	0.69054 (7)	0.51723 (15)	0.17669 (8)	0.0189 (3)	
C13	0.63915 (7)	0.42696 (16)	0.11247 (8)	0.0204 (3)	
H13A	0.6548	0.3671	0.0804	0.024*	
C14	0.56517 (7)	0.42431 (16)	0.09521 (8)	0.0197 (3)	
H14A	0.5304	0.3604	0.0521	0.024*	
C15	0.76862 (7)	0.52180 (16)	0.19011 (8)	0.0206 (3)	
C16	0.89312 (7)	0.58733 (19)	0.27969 (9)	0.0289 (3)	
H16A	0.9252	0.6008	0.3375	0.043*	
H16B	0.8997	0.6789	0.2512	0.043*	
H16C	0.9066	0.4882	0.2619	0.043*	

### Atomic displacement parameters (Å^2^)

**Table:**

	*U*^11^	*U*^22^	*U*^33^	*U*^12^	*U*^13^	*U*^23^
O1	0.0154 (5)	0.0204 (5)	0.0315 (5)	−0.0001 (4)	0.0110 (4)	0.0023 (4)
O2	0.0175 (5)	0.0207 (5)	0.0339 (5)	0.0015 (4)	0.0119 (4)	−0.0003 (4)
O3	0.0263 (5)	0.0246 (5)	0.0434 (6)	−0.0047 (4)	0.0214 (5)	−0.0125 (5)
O4	0.0193 (5)	0.0267 (5)	0.0272 (5)	0.0007 (4)	0.0142 (4)	0.0032 (4)
O5	0.0147 (4)	0.0331 (6)	0.0258 (5)	−0.0010 (4)	0.0084 (4)	−0.0031 (4)
O6	0.0215 (5)	0.0371 (6)	0.0365 (6)	−0.0022 (4)	0.0170 (5)	−0.0112 (5)
N1	0.0128 (5)	0.0225 (6)	0.0228 (6)	0.0013 (4)	0.0075 (4)	−0.0013 (5)
C1	0.0149 (6)	0.0256 (7)	0.0309 (7)	−0.0014 (5)	0.0107 (6)	0.0017 (6)
C2	0.0181 (6)	0.0199 (7)	0.0176 (6)	−0.0011 (5)	0.0082 (5)	−0.0019 (5)
C3	0.0160 (6)	0.0225 (7)	0.0158 (6)	0.0004 (5)	0.0073 (5)	−0.0002 (5)
C4	0.0176 (6)	0.0199 (7)	0.0163 (6)	0.0025 (5)	0.0080 (5)	0.0000 (5)
C5	0.0181 (6)	0.0205 (7)	0.0158 (6)	−0.0007 (5)	0.0089 (5)	−0.0010 (5)
C6	0.0173 (6)	0.0248 (7)	0.0152 (6)	0.0022 (5)	0.0076 (5)	−0.0008 (5)
C7	0.0192 (6)	0.0205 (7)	0.0230 (7)	0.0035 (5)	0.0096 (5)	0.0011 (5)
C8	0.0202 (6)	0.0203 (7)	0.0213 (7)	−0.0008 (5)	0.0099 (5)	0.0005 (5)
C9	0.0158 (6)	0.0204 (7)	0.0198 (7)	0.0026 (5)	0.0081 (5)	0.0036 (5)
C10	0.0201 (6)	0.0245 (7)	0.0184 (6)	0.0038 (5)	0.0100 (5)	0.0004 (5)
C11	0.0178 (6)	0.0228 (7)	0.0172 (6)	0.0009 (5)	0.0064 (5)	0.0007 (5)
C12	0.0160 (6)	0.0188 (7)	0.0214 (7)	0.0026 (5)	0.0086 (5)	0.0034 (5)
C13	0.0205 (6)	0.0189 (7)	0.0235 (7)	0.0036 (5)	0.0119 (5)	−0.0001 (5)
C14	0.0182 (6)	0.0193 (7)	0.0199 (6)	0.0006 (5)	0.0077 (5)	−0.0002 (5)
C15	0.0187 (6)	0.0164 (7)	0.0258 (7)	0.0006 (5)	0.0099 (6)	−0.0001 (5)
C16	0.0155 (6)	0.0345 (8)	0.0337 (8)	−0.0018 (6)	0.0094 (6)	−0.0025 (7)

### Geometric parameters (Å, º)

**Table:**

O1—C2	1.3331 (16)	C6—C9	1.4906 (17)
O1—C1	1.4504 (14)	C7—C8	1.3883 (18)
O2—C2	1.2030 (16)	C7—H7A	0.9500
O3—N1	1.2235 (14)	C8—H8A	0.9500
O4—N1	1.2240 (14)	C9—C14	1.3905 (19)
O5—C15	1.3364 (16)	C9—C10	1.3932 (18)
O5—C16	1.4461 (15)	C10—C11	1.3853 (17)
O6—C15	1.2030 (17)	C10—H10A	0.9500
N1—C5	1.4716 (17)	C11—C12	1.3894 (18)
C1—H1A	0.9800	C11—H11A	0.9500
C1—H1B	0.9800	C12—C13	1.3905 (18)
C1—H1C	0.9800	C12—C15	1.4891 (18)
C2—C3	1.4913 (17)	C13—C14	1.3841 (17)
C3—C4	1.3866 (18)	C13—H13A	0.9500
C3—C8	1.3902 (18)	C14—H14A	0.9500
C4—C5	1.3828 (17)	C16—H16A	0.9800
C4—H4A	0.9500	C16—H16B	0.9800
C5—C6	1.3928 (18)	C16—H16C	0.9800
C6—C7	1.3942 (19)		
			
C2—O1—C1	114.26 (10)	C7—C8—H8A	120.1
C15—O5—C16	115.38 (11)	C3—C8—H8A	120.1
O3—N1—O4	124.13 (11)	C14—C9—C10	119.29 (12)
O3—N1—C5	117.62 (10)	C14—C9—C6	119.63 (11)
O4—N1—C5	118.25 (10)	C10—C9—C6	121.06 (12)
O1—C1—H1A	109.5	C11—C10—C9	120.51 (12)
O1—C1—H1B	109.5	C11—C10—H10A	119.7
H1A—C1—H1B	109.5	C9—C10—H10A	119.7
O1—C1—H1C	109.5	C10—C11—C12	119.77 (12)
H1A—C1—H1C	109.5	C10—C11—H11A	120.1
H1B—C1—H1C	109.5	C12—C11—H11A	120.1
O2—C2—O1	123.70 (11)	C11—C12—C13	120.00 (12)
O2—C2—C3	123.34 (12)	C11—C12—C15	122.29 (12)
O1—C2—C3	112.95 (11)	C13—C12—C15	117.66 (12)
C4—C3—C8	120.06 (12)	C14—C13—C12	119.98 (12)
C4—C3—C2	117.06 (11)	C14—C13—H13A	120.0
C8—C3—C2	122.87 (11)	C12—C13—H13A	120.0
C5—C4—C3	118.53 (11)	C13—C14—C9	120.40 (12)
C5—C4—H4A	120.7	C13—C14—H14A	119.8
C3—C4—H4A	120.7	C9—C14—H14A	119.8
C4—C5—C6	123.42 (12)	O6—C15—O5	123.68 (12)
C4—C5—N1	116.52 (11)	O6—C15—C12	123.98 (12)
C6—C5—N1	120.03 (11)	O5—C15—C12	112.34 (12)
C5—C6—C7	116.40 (12)	O5—C16—H16A	109.5
C5—C6—C9	123.47 (12)	O5—C16—H16B	109.5
C7—C6—C9	120.11 (11)	H16A—C16—H16B	109.5
C8—C7—C6	121.69 (12)	O5—C16—H16C	109.5
C8—C7—H7A	119.2	H16A—C16—H16C	109.5
C6—C7—H7A	119.2	H16B—C16—H16C	109.5
C7—C8—C3	119.86 (12)		
			
C1—O1—C2—O2	−1.86 (18)	C2—C3—C8—C7	−179.73 (11)
C1—O1—C2—C3	177.73 (10)	C5—C6—C9—C14	−125.48 (14)
O2—C2—C3—C4	4.93 (19)	C7—C6—C9—C14	55.96 (17)
O1—C2—C3—C4	−174.66 (11)	C5—C6—C9—C10	56.00 (18)
O2—C2—C3—C8	−175.54 (13)	C7—C6—C9—C10	−122.56 (14)
O1—C2—C3—C8	4.87 (18)	C14—C9—C10—C11	0.48 (19)
C8—C3—C4—C5	1.65 (19)	C6—C9—C10—C11	179.01 (12)
C2—C3—C4—C5	−178.80 (11)	C9—C10—C11—C12	1.38 (19)
C3—C4—C5—C6	−1.81 (19)	C10—C11—C12—C13	−1.77 (19)
C3—C4—C5—N1	176.20 (11)	C10—C11—C12—C15	175.57 (12)
O3—N1—C5—C4	48.64 (15)	C11—C12—C13—C14	0.28 (19)
O4—N1—C5—C4	−130.48 (12)	C15—C12—C13—C14	−177.17 (12)
O3—N1—C5—C6	−133.27 (12)	C12—C13—C14—C9	1.60 (19)
O4—N1—C5—C6	47.60 (16)	C10—C9—C14—C13	−1.98 (19)
C4—C5—C6—C7	0.46 (19)	C6—C9—C14—C13	179.47 (12)
N1—C5—C6—C7	−177.48 (11)	C16—O5—C15—O6	0.21 (19)
C4—C5—C6—C9	−178.14 (12)	C16—O5—C15—C12	−179.65 (11)
N1—C5—C6—C9	3.91 (19)	C11—C12—C15—O6	−162.42 (13)
C5—C6—C7—C8	1.06 (19)	C13—C12—C15—O6	15.0 (2)
C9—C6—C7—C8	179.72 (12)	C11—C12—C15—O5	17.44 (18)
C6—C7—C8—C3	−1.2 (2)	C13—C12—C15—O5	−165.16 (12)
C4—C3—C8—C7	−0.20 (19)		

### Hydrogen-bond geometry (Å, º)

**Table:**

*D*—H···*A*	*D*—H	H···*A*	*D*···*A*	*D*—H···*A*
C4—H4*A*···O6^i^	0.95	2.50	3.3405 (16)	148
C13—H13*A*···O2^ii^	0.95	2.39	3.2435 (16)	150
C14—H14*A*···O4^iii^	0.95	2.59	3.3954 (16)	143

Symmetry codes: (i) *x*−1/2, *y*+1/2, *z*; (ii) *x*+1/2, *y*−1/2, *z*; (iii) −*x*+1, −*y*+1, −*z*.
